# Long-term mortality after coronary surgery in women patients depend on diabetes and age

**DOI:** 10.1093/icvts/ivaf069

**Published:** 2025-03-26

**Authors:** Victor Dayan, Juan Andres Montero, Maximiliano Hernandez, Carolina Sosa, Santiago Cubas, Stefano Urso, Rafael Sadaba, Nick Freemantle

**Affiliations:** Hospital de Clinicas, Universidad de la Republica, Montevideo, Uruguay; Hospital de Clinicas, Universidad de la Republica, Montevideo, Uruguay; Instituto Nacional de Cirugia Cardiaca, Montevideo, Uruguay; Hospital de Clinicas, Universidad de la Republica, Montevideo, Uruguay; Hospital de Clinicas, Universidad de la Republica, Montevideo, Uruguay; Cardiac Surgery Department, Hospital Universitario Dr Negrín, Las Palmas de Gran Canaria, Spain; Cardiac Surgery Department, Hospital Universitario de Navarra, Pamplona, Spain; Comprehensive Clinical Trials Unit, Institute of Clinical Trials and Methodology, University College London, London, UK

**Keywords:** CABG, survival, gender

## Abstract

**OBJECTIVES:**

There is general consensus of the higher short-term risk in women after coronary artery bypass grafts (CABG), nonetheless, long-term survival is a matter of debate. We aimed to compare in a national database with over 10 years of follow-up long-term survival in women versus men and its interaction with diabetes and age.

**METHODS:**

This is a national retrospective cohort study from Uruguay. Patients were included if they underwent isolated CABG between 1 January 2002 and 31 December 2022. The primary outcome was survival. The secondary outcome was a composite of operative mortality, postoperative stroke, deep sternal wound infection and kidney failure requiring dialysis. Interaction of age and diabetes was explored in the survival analysis after adjusting for baseline characteristics.

**RESULTS:**

During the included study period, 21 959 patients (5778 were women) underwent isolated CABG in Uruguay. Among people with diabetes, women had worse survival, while no differences between gender were found in the non-diabetic population. Survival at 1 year after CABG was significantly lower in women (hazard ratio (HR) = 1.20; 95% confidence interval (CI): 1.07, 1.35; *P* = 0.002). Survival after 1-year was higher in women (*P* < 0.001). Absence of diabetes improved survival (HR = 0.83; 95% CI: 0.77, 0.89; *P* < 0.001), while presence of diabetes made survival between men and women similar (HR = 1.00; 95% CI: 0.92, 1.09; *P* = 0.946). Interaction between age and gender showed that women older than 60 years old had better survival than men. Composite outcome was worse in women (OR = 1.47; 95% CI: 1.24, 1.75).

**CONCLUSIONS:**

Women patients have worse overall mortality but better long-term survival than men. Diabetes and age have significant interaction with the long-term outcomes. Better survival is seen in women older than 60 years old.

## Graphical abstract

**Figure:**
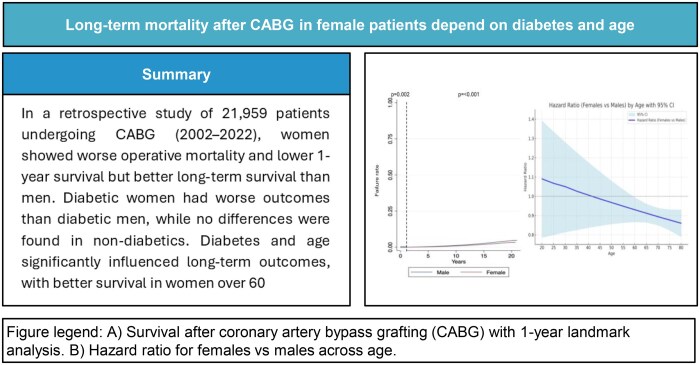

## INTRODUCTION

Cardiovascular heart disease is the main cause of death of women worldwide, being responsible for one in every three deaths [1]. Coronary artery disease, along with cerebrovascular disease, is the main determinant of death. While many studies have addressed the higher risk of short-term operative outcomes in women patients after coronary artery bypass grafts (CABG) [2–4], the long-term outcome has been more controversial [5–7]. Guru *et al.* [7] have shown that long-term mortality in women was numerically lower than their men counterpart. Women patients have also been shown to have worse short-term outcome after percutaneous coronary interventions [8]. Different possible explanations have been proposed to explain such a discrepancy in long-term outcomes: sex-based disparities (access barriers and biases for women but not men) higher incidence of diabetes, hypertension, older ager, worse coronary artery anatomy and surgery at a later stage in the clinical course of the CAD [9].

Although diabetes is a well-known variable associated with for worse survival after CABG [10, 11], most studies that evaluate the differential survival of women patients compared to men only adjust for presence or absence of diabetes, ignoring the wide range of risk faced by people with different stages of the disease and do not calculate its interaction or stratify outcomes according to its presence.

We compared the long-term survival of women versus men patients after isolated CABG and the impact of diabetes on this outcome.

## MATERIALS AND METHODS

This is a retrospective cohort study based on the adult cardiac surgery database of the National Resources Fund of Uruguay. The study has been approved by the Institutional Review Board from the British Hospital, Montevideo, Uruguay, which waived the need for individual patient consent.

The NRF is the governmental agency in charge of the financing of 100% cardiac surgery procedures in Uruguay. To get reimbursement, the NRF required completion of baseline, operative and postoperative variables. Therefore, the NRF is a compulsory prospectively maintained and audited database.

Vital status follow-up is performed through the Epidemiology Department of the Ministry of Health, using statutory mortality reporting data.

### Population

Patients were included if they underwent isolated CABG between 1 January 2002 and 31 December 2022. Patients requiring resuscitation prior to surgery, emergency procedures or reoperation were excluded.

The primary outcome was survival. The secondary outcome was a composite of operative mortality, postoperative stroke, deep sternal wound infection (DSWI) and kidney failure requiring dialysis. Individual components of the secondary composite outcome were assessed independently, along with prolonged inotropic support, prolonged mechanical ventilation and postoperative atrial fibrillation.

### Definitions

Operative mortality: in hospital death anytime during index hospitalization or within 30 days of CABG after discharge. Postoperative stroke: during the first 30 days after surgery. Prolonged inotropic support: lasting more than 24 h after surgery. Prolonged mechanical ventilation: lasting more than 12 h after surgery. Postoperative atrial fibrillation: before hospital discharge. Complete revascularization: at least one bypass in a major coronary system (left anterior descending artery, circumflex and right coronary) with a stenosis >50%. Arterial revascularization (AR): an additional arterial graft besides left internal mammary artery to the left anterior descending artery.

### Statistics

Shapiro–Wilk test for normal data was performed for continuous variables. Since all were non-normally distributed, continuous variables were reported as median and interquartile range, and comparison between groups was performed using Mann–Whitney *U*-test. Categorical variables were presented as absolute values (%) and compared using Fisher’s exact test.

Risk for the composite (and its individual outcomes) outcome was adjusted according to baseline and intraoperative variables that were significantly different between groups.

Median follow-up time was calculated using inverse Kaplan–Meier. Survival was assessed with Kaplan–Meier and comparison among groups with log-rank test. Proportional hazard assumption was tested based on Schoenfeld residuals and graphically assessed (log–log curves) (Supplementary Material, Fig. S1). Since gender violated proportional hazard assumption (rho −0.03706, *P* = 0.0007), landmark analysis was performed. A fixed-time landmark approach was used at 1 year post-surgery to divide the follow-up period into two distinct phases: early phase (<1 year post-CABG: includes all patients from the time of surgery to the 1-year mark, patients who survived more than 1 year were censored) and late phase (>1 year post-CABG: includes only those patients who survived beyond the 1-year landmark). The rationale for choosing the 1-year cutoff was based on: visual inspection of Kaplan–Meier survival curves, which suggested a change in survival trends around this time; clinical relevance, as the majority of operative and perioperative events occur within the first year after CABG; ensuring the proportional hazards assumption held true within each phase, as tested post-landmarking.

For survival analysis beyond 1 year, we re-tested the proportional hazards assumption and performed Cox regression only on patients who survived past the landmark point, thereby preventing immortal time bias.

Univariate adjustment was performed with potential confounders (those covariates that were significantly different among groups) through univariate regression, and if significant, they were included in the final multivariable logistic model. Univariate confounding and effect modification were assessed using Mantel–Haenszel stratification, and multivariable adjustment was performed using logistic regression. Only variables with a *P* < 0.1 in the univariable analysis were included in the multivariable model. Stepwise regression approach was the regression procedure performed in the multivariable analysis (for either logistic regression or Cox regression). Breslow method was used for ties. Likelihood ratio test was used to test interaction between variables that are significant in the final model (multivariable model). If significance in the interaction was found, the regression model was re-run with the interaction term. Interaction in the cox multivariable regression model was evaluated with likelihood ratio test for the variables found to be significant in the multivariable analysis. If present, the interaction term was included in the final model. Baseline confounders included diabetes, smoking, stroke, creatinine, left ventricular ejection fraction (LVEF), hypertension, dyslipidaemia. In order to evaluate the relative effect of gender across age, this variable was modelled as a continuous variable. The results were reported as hazard ratios (HRs) with 95% confidence intervals (CIs), representing the relative hazard for women compared to males across different ages. To visualize the change in HR across age, we used the margins command in Stata to compute predicted HRs and their 95% CIs over a range of ages (20–80 years, at 5-year intervals). The predicted HRs were plotted as a function of age, with the CIs represented as shaded areas to provide a clear depiction of the precision of estimates at different age points.

## RESULTS

### Baseline characteristics

During the included study period, 21 959 patients underwent isolated CABG in Uruguay. Of them 5778 were women (26.3%) and 16 181 were men (73.7%). The median age of the whole population was 65 (58–72) years old, 33.2% of patients had diabetes and three-vessel disease was present in 79.4%. Women patients were older, with a higher incidence of diabetes, dyslipidaemia, hypertension and unstable angina. By contrast, male patients had higher incidence of smoking, three-vessel disease, worse baseline creatinine and worse LVEF (Table 1). Vital status follow-up was available in 99.4%. Median follow-up was 11.4 years (11.1 years for men and 11.6 years for women). Median survival was 13.9 years (95% CI: 13.7, 14.1).

**Table 1:** Patients baseline characteristics (*n* = 21 959)

	Total	Women (5778)	Men (16 181)	*P*-value
Age (years), median (IQR)	65 (58–72)	68 (60–74)	64 (57–71)	<0.001
Diabetes, *n* (%)	7292 (33.2)	2299 (39.8)	4993 (30.9)	<0.001
Stroke, *n* (%)	490 (2.2)	150 (2.6)	340 (2.1)	0.029
Dyslipidaemia, *n* (%)	12 340 (56.2)	3327 (57.6)	9013 (55.7)	0.013
Smoker, *n* (%)	6663 (30.3)	1213 (21.0)	5450 (33.7)	<0.001
Hypertension, *n* (%)	14 538 (66.2)	4215 (72.9)	10 323 (63.8)	<0.001
Previous AMI, *n* (%)	4897 (22.3)	1258 (21.8)	3639 (22.5)	0.261
Weight (kg), median (IQR)	80 (70–90)	70 (62–80)	81 (73–90)	<0.001
Height (cm), median (IQR)	170 (162–175)	160 (155–164)	171 (168–176)	<0.001
Creatinine (mg/dl), median (IQR)	1.00 (0.85–1.20)	0.90 (0.76–1.08)	1.02 (0.89–1.20)	<0.001
LVEF (%), median (IQR)	55 (45–60)	58 (47–60)	55 (45–60)	<0.001
Previous PCI, *n* (%)	5780 (26.3)	1420 (24.6)	4360 (27.0)	<0.001
Unstable angina, *n* (%)	11 070 (50.4)	3034 (52.5)	8036 (49.7)	<0.001
Three-vessel disease, *n* (%)	17 438 (79.4)	4401 (76.2)	13 037 (80.6)	<0.001

AMI: acute myocardial infarction; LVEF: left ventricular ejection fraction.

### Procedural characteristics

Off-pump CABG (OPCABG) was performed in 5750 (26%) of cases, complete revascularization was achieved in 12 943 (58%) and AR was performed in 2761 (13%). Women patients had higher incidence of OPCABG, higher intraoperative glycaemia and lower incidence of AR (Table 2).

**Table 2:** Intraoperative variables (*n* = 21 959)

	Total	Women (5778)	Men (16 181)	*P*-value
OPCABG, *n* (%)	5750 (26.2)	1723 (29.8)	4027 (24.9)	<0.001
CPB time (min), median (IQR)	85 (70–103)	84 (67–100)	85 (70–104)	<0.001
AXC time (min), median (IQR)	48 (37–60)	46 (35–58)	49 (38–61)	<0.001
IOP glycaemia (mg/dl), median (IQR)	1.70 (1.40–2.04)	1.79 (1.43–2.18)	1.69 (1.40–2.00)	<0.001
Arterial revascularization, *n* (%)	2761 (12.6)	431 (7.5)	2330 (14.4)	<0.001
RIMA, *n* (%)	2162 (9.9)	296 (5.1)	1866 (11.5)	<0.001
Radial, *n* (%)	806 (3.7)	174 (3.0)	632 (3.9)	0.002
Number of bypass	2 (2–3)	2 (2–3)	2 (2–3)	<0.001
1	1928 (8.8)	697 (12.1)	1231 (7.6)	
2	1017 (45.6)	2631 (45.5)	7386 (45.7)	
3 or +	1001 (45.5)	2447 (42.4)	7554 (46.7)	
CR (%)	12 943 (58.9)	3370 (58.3)	9573 (59.2)	0.267

AXC: aortic cross-clamp; CPB: cardiopulmonary bypass; CR: complete revascularization; IOP: highest intraoperative; RIMA: right internal mammary artery.

### Postoperative outcomes

The un-adjusted composite outcome was more frequent in women patients (7.5% vs 5.6%; *P* < 0.001). Similarly, operative mortality (4.9% vs 3.5; *P* < 0.001) and stroke (1.5% vs 1.2%; *P* = 0.024) were higher in women patients, while no difference were found in requirement of postoperative dialysis (Table 3). DSWI, hospital stay and postoperative glycaemia were higher in women patients.

**Table 3:** Postoperative outcomes and women risk on composite outcome (*n* = 21 959)

	Total	Women (5778)	Men (16 181)	*P*-value	OR_adj_ (95% CI)	*P*_adj_
Composite outcome	1339 (6.1)	433 (7.5)	906 (5.6)	<0.001	1.47 (1.24, 1.75)	<0.001
Operative mortality	851 (3.9)	281 (4.9)	570 (3.5)	<0.001	1.54 (1.24, 1.90)	<0.001
Stroke	276 (1.3)	89 (1.5)	187 (1.2)	0.027	1.03 (0.71, 1.48)	0.883
Dialysis	194 (0.9)	46 (0.8)	148 (0.9)	0.409	1.07 (0.68, 1.67)	0.775
DSWI	196 (0.9)	66 (1.1)	130 (0.8)	0.019	1.82 (1.23, 2.69)	0.003
Hospital stay, days, median (IQR)	8 (7–11)	8 (7–11)	8 (7–10)	<0.001		
ICU stay (days), median (IQR)	2 (2–3)	2 (2–3)	2 (2–3)	0.229		
PO glycaemia, mg/dl, median (IQR)	1.85 (1.48–2.26)	1.90 (1.50–2.35)	1.83 (1.47–2.23)	<0.001		
Prolonged IS	9268 (42.2)	2320 (40.2)	6948 (42.9)	<0.001	0.89 (0.82, 0.97)	0.009
Prolonged MV	6847 (31.2)	1789 (31.0)	5058 (31.3)	0.676	1.02 (0.93, 1.12)	0.715

Adj: odds ratio for women gender derived from multivariable logistic regression; DSWI: deep sternal wound infection; ICU: intensive care unit; IS: inotropic support; MV: mechanical ventilation; PO: highest postoperative.

After multivariable logistic regression, women gender was an independent predictor for the composite outcome (OR = 1.47; 95% CI: 1.24, 1.75; *P* < 0.001). Such a higher risk of the composite was derived mainly from a significantly higher risk for operative mortality (OR = 1.54; 95% CI: 1.24, 1.90; *P* < 001). Similarly, women gender was an independent predictor for DSWI (OR = 1.82; 95% CI: 1.23, 2.69; *P* < 0.003). On the contrary, women gender protected from prolonged inotropic support (OR = 0.89; 95% CI: 0.82, 0.97; *P* = 0.009).

### Survival

#### Un-adjusted analysis

**Figure 1:**
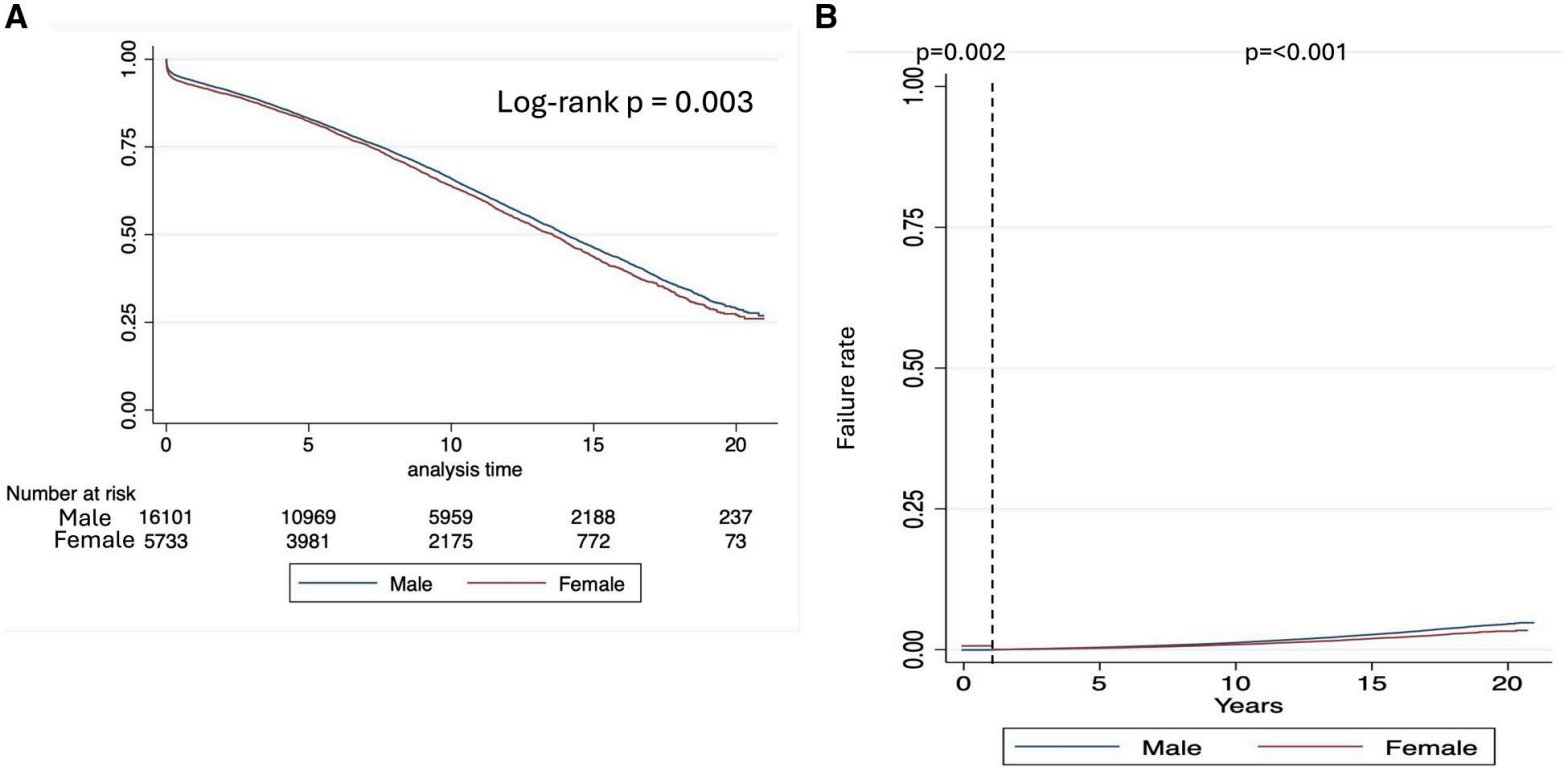
Kaplan–Meier survival plot. (**A**) Overall analysis and (**B**) 1-year landmark analysis

**Figure 2:**
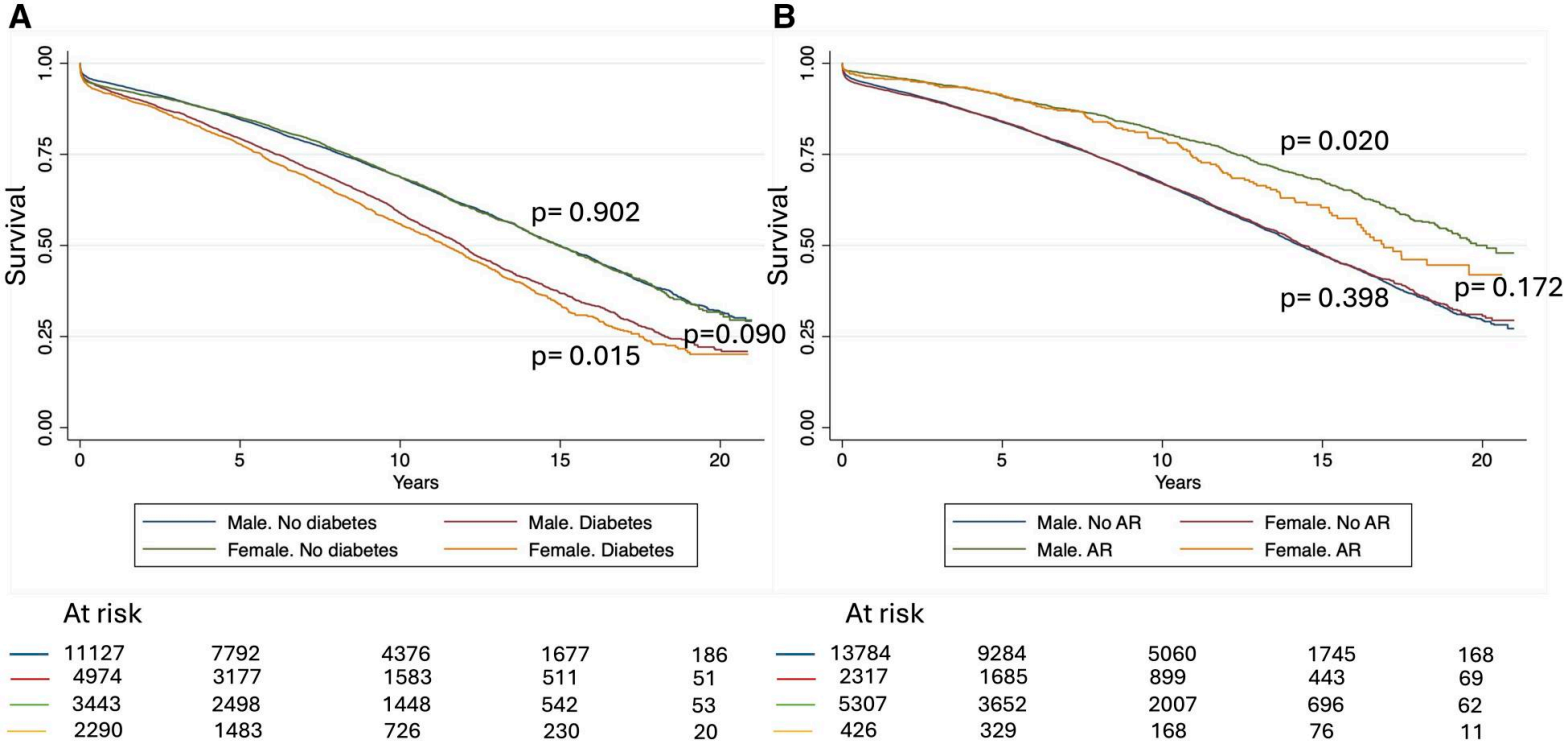
Survival after coronary artery bypass grafting stratified by gender and (**A**) diabetes or (**B**) arterial revascularization

The median survival of the overall population was 13.9 years (95% CI: 13.7, 14.1). Un-adjusted survival was lower in women patients (Fig. 1A) and did not change when different eras were analysed (Supplementary Material, Fig. S2). Un-adjusted subgroup analysis is shown in Supplementary Material, Fig. S3. Among patients with diabetes, women had worse survival, while no differences between gender were found in the non-diabetic population (Fig. 2A). When survival was stratified by gender and AR, no differences were found among patients who did not receive AR (*P* = 0.398), but worse survival was noted in women (versus men) patients who received AR (*P* = 0.020) (Fig. 2B). In this strata (AR) women and men survival curves start to diverge after ∼7 years.

Univariate predictors on survival are shown in Supplementary Material, Table S1. Significant variables were included in the final multivariable model. Since the constant proportional hazard assumption for gender was not met, landmark analysis was performed (Supplementary Material, Table S2).

#### One-year follow-up adjusted analysis

Adjusted 1-year survival analysis was lower in women patients (Fig. 1B). No interaction was found between gender and any of the covariates. Survival at 1 year after CABG was significantly lower in women patients (HR = 1.20; 95% CI: 1.07, 1.35; *P* = 0.002) (Table 4).

**Table 4:** Multivariable predictors of survival (*n* = 21 258)

	One year (*n* = 21 258)		Over 1 year (19 857)		
	HR (95% CI)	*P*-value	HR (95% CI)	*P*-value	*P*_int_
Women	1.20 (1.07, 1.35)	0.002			<0.001
Diabetic			1.00 (0.92, 1.09)	0.946	
Non diabetic			0.83 (0.77, 0.89)	<0.001	
Age	1.07 (1.06, 1.07)	<0.001	1.06 (1.06, 1.06)	<0.001	
Stroke	1.26 (0.94, 1.21)	0.119	1.49 (1.31, 1.70)	<0.001	
Dyslipidaemia	0.87 (0.78, 0.96)	0.008	0.93 (0.88, 0.97)	0.002	
Smoker	1.36 (1.21, 1.54)	<0.001	1.28 (1.21, 1.36)	<0.001	
Hypertension	1.08 (0.96, 1.21)	0.178	1.17 (1.11, 1.25)	<0.001	
Creatinine	1.20 (1.17, 1.23)	<0.001	1.15 (1.13, 1.17)	<0.001	
LVEF	0.97 (0.96, 0.97)	<0.001	0.98 (0.98, 0.99)	<0.001	
Three-vessel disease	1.13 (0.98, 1.30)	0.103	1.06 (1.00, 1.13)	0.051	
AR	0.90 (0.73, 1.11)	0.334	0.89 (0.81, 0.97)	0.010	

LVEF: left ventricular ejection fraction; AR: arterial revascularization.

#### Over 1 year follow-up adjusted analysis

**Figure 3:**
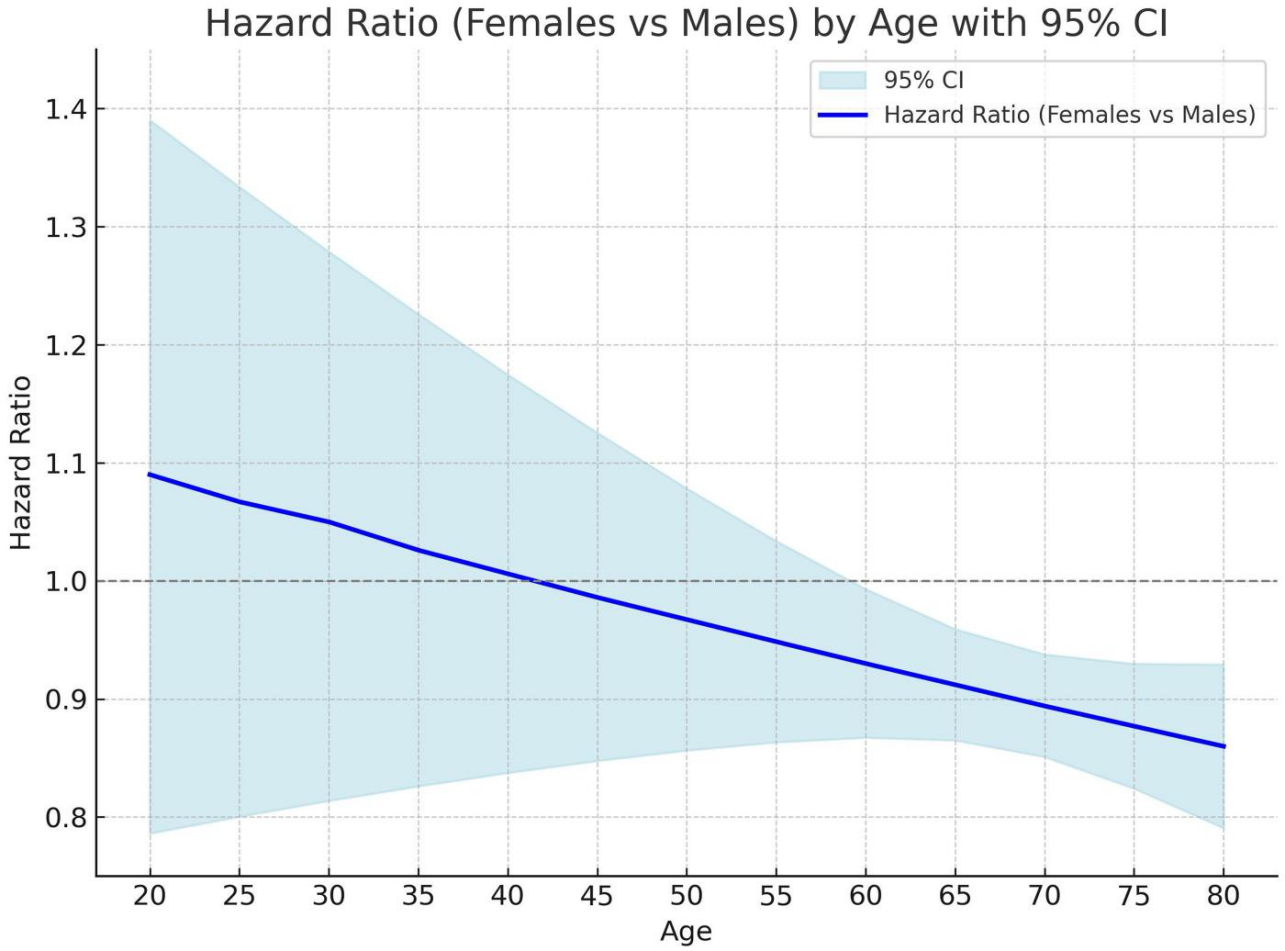
Hazard ratio (HR) by age. Continuous line is the HR and the light blue bands represent the 95% CI

Survival after 1-year was higher in women patients (Fig. 1B). A significant interaction was found between gender and diabetes (pint < 0.001). Absence of diabetes improved survival in women compared to men (HR = 0.83; 95% CI: 0.77, 0.89; *P* < 0.001), while presence of diabetes made survival between men and women similar (HR = 1.00; 95% CI: 0.92, 1.09; *P* = 0.946) (Table 4). Interaction between age and gender showed that survival was worse in younger women but better than men in older women (Fig. 3). This change in survival occurred approximately at 60 years old.

## DISCUSSION

There are no large long-term follow-up studies that evaluate the interaction of diabetes and age on outcomes after CABG in women patients. Our national cohort shows that women have worse overall survival. This is especially true in diabetic women. On the contrary, among non-diabetic patients’, women have better survival than males. The composite outcome was significantly higher in women patients compared to men. This outcome was driven mainly by higher postoperative mortality. Regarding age, we were able to show that the relative survival of women compared to men varied with age and that 60 years old was the turning point.

Large prospective epidemiological cohorts have shown that the incidence of ischaemic heart disease is lower and primary prevention therapy is higher in women patients [12]. Although the incidence of CABG is lower in women patients, recurrence of cardiovascular events and short-term mortality after an event was lower in women patients [12]. In the ISCHEMIA trial, women were less likely to undergo revascularization mainly because of the absence of obstructive coronary lesions [13]. Although no differences were found in long-term all-cause mortality between men and women patients, a careful analysis of Kaplan–Meier survival curves shows that curves diverge after 3 years of follow-up [13]. Kogan *et al.* [11] found mortality to increase in diabetic patients compared to non-diabetic only after 3 years of CABG. Similar to previous reports, our national women cohort consisted of patients with a higher incidence of baseline cardiovascular risk factor such as older age, diabetes, hypertension and dyslipidaemia but a lower level of baseline creatinine values, smoker incidence and better LVEF [2, 6, 14]. Attia *et al.* [14] report gender differences in the institutional database of the Cleveland Clinic. Similar to our findings, in their cohort, the incidence of AR was lower and OPCABG higher in women. Postoperative outcomes were also worse in women with higher operative mortality compared to their male counterparts. In contrast to our findings, long-term survival in women patients was worse in the cohort presented by Attia *et al.* When we compare the survival curves of Attia *et al.* and our findings, we see that the 10-year survival of women is similar in both cohorts, but the 10-year survival in men is much higher in the Attia *et al.* report. This may explain the different findings of both registries.

In contrast to large US registries, the proportion of diabetes in women patients who undergo CABG in our country was lower (39.8% vs 56.3%) albeit significantly higher than men [6].

After adjustment for baseline risk factors, women patients were at increased risk for the composite outcome. This was driven by an increased risk in operative mortality, which was 54% higher. Among other postoperative outcomes, we found a significantly higher risk for DSWI and prolonged inotropic support. Interestingly, not only diabetes was more prevalent in women patients, but also intra- and postoperative glycaemia was higher in women patients as well. Knapik *et al.* [15] have shown that CEC increases intraoperative glycaemia, and that women patients are a variables associated with for difficult control of intra and postoperative glycaemic values. Previous studies have shown an independent association between these and postoperative outcomes [16].

Un-adjusted overall survival was worse in women patients. Diabetes was a significant modifier of the effect of gender on long-term mortality after CABG. When long-term mortality was evaluated after accounting for baseline characteristics, we found that women were at lower risk of mortality in the non-diabetic population, while no differences were found in diabetics. The impact of diabetes on the adverse outcome in women patients is evident after 3–5 years after surgery. While previous studies have shown an increased risk of long-term mortality in women patients [14], others have failed to do so [17]. AR has shown to improve survival in retrospective studies in males, but not in women [6, 14]. In our study, women survival was similar to males in patients who did not undergo AR. In patients who underwent AR, curves started to diverge after 7 years of surgery approximately. Similarly, in a retrospective analysis of the New York State Cardiac Surgery Reporting System of all patients undergoing CABG, in contrast to men patients, women did not derive a mortality benefit with AR [18].

Most retrospective studies agree that women patients are at increased risk of short-term mortality after CABG, while long-term mortality is controversial. The impact of age in the relative survival compared to men has been scarcely evaluated. Lemaire *et al.* [19] in a cohort of >70-year-old patients reported lower survival in women with increasing age. Ter Woorst *et al.* [20] in a single-centre study showed that women had worse survival only when younger than 70 years old; older women had similar survival than men. In contrast to previous studies, our data show that older women do better in the long term compared to men and that this significant change occurs after 60 years old. This finding could be due to selection bias, and only those women with better coronary arteries and lower risk profile may have been operated, which explains this difference with men. On the contrary, women globally have longer life expectancy than men, which means that our findings may just be replicating life expectancy.

There is great debate as to the real cause for the differences in outcomes between men and women patients. Is it biologically driven or practice driven? Data from the USA have shown than women patients undergo surgery most frequently in low-quality hospitals. This is an important un-measured confounder and equity criteria that needs to be addressed to ensure the best overall outcomes. Considering the higher risk of these patients, women patients should be considered as high-risk patients during treatment planning and referred to centres, which may ensure equitable care [21].

### Limitations

Inherent to retrospective studies, we need to highlight the limitation due to selection bias. In this sense, women with poor coronary arteries may be rejected for surgery compared to men and therefore affect the real outcomes. Even though extensive adjustment was performed, we cannot rule out residual confounding, which is very difficult to adjust for and which may explain the differential outcomes. The outcomes presented cannot be generalized to other countries or populations due to the lack of assessment of other factors such as socioeconomic variables, which are known to influence outcomes.

## CONCLUSION

Overall un-adjusted survival was worse in women. Our study is the first to highlight the interaction of diabetes on gender in long-term outcomes. Considering <30% of participants of most revascularization trials are women, such findings may not be applicable to women patients.

## Supplementary Material

ivaf069_Supplementary_Data.docx

## Data Availability

Data availability will be considered by the Ministry of Health in case of requests.

## Notes

Presented and selected as best coronary abstract at the 2024 EACTS/STS/LACES LATAM meeting 6th December 2024, Buenos Aires, Argentina.

## ABBREVIATIONS

AR: Arterial revascularization
CABG: Coronary artery bypass grafts
CI: Confidence interval
DWSI: Deep sternal wound infection
HR: Hazard ratio
LVEF: Left ventricular ejection fraction
OPCABG: Off-pump CABG
