# Supplementary material for: Long-term mortality after coronary surgery in women patients depend on diabetes and age
Source: Interdiscip Cardiovasc Thorac Surg. 2025 Mar 26;40(4):ivaf069. doi: 10.1093/icvts/ivaf069 (PMC11954628; doi:10.1093/icvts/ivaf069)
Supplement: ivaf069_Supplementary_Data.docx [file ivaf069_supplementary_data.docx]

|  | HR (95%CI) | p |
| --- | --- | --- |
| Female | 1.07 (1.02, 1.12) | 0.003 |
| Age | 1.06 (1.05, 1.06) | <0.001 |
| Diabetes | 1.45 (1.38, 1.51) | <0.001 |
| Stroke | 1.67 (1.49, 1.88) | <0.001 |
| Dislipidemia | 0.88 (0.85, 0.92) | <0.001 |
| Smoker | 0.94 (0.90, 0.99) | 0.010 |
| Hypertension | 1.24 (1.18, 1.30) | <0.001 |
| Weight | 0.99 (0.99, 1.00) | <0.001 |
| Height | 0.99 (0.99, 1.00) | 0.080 |
| Creatinine | 1.17 (1.15, 1.19) | <0.001 |
| LVEF | 0.99 (0.98, 0.99) | <0.001 |
| Previous PCI | 1.00 (0.96, 1.05) | 0.939 |
| Unstable angina | 0.96 (0.92, 1.00) | 0.052 |
| 3-vessel disease | 1.22 (1.15, 1.29) | <0.001 |
| Arterial revascularization | 0.55 (0.51, 0.59) | <0.001 |
| OnPCABG | 0.97 (0.93, 1.01) | 0.159 |
| Number of bypass | 0.92 (0.89, 0.95) | <0.001 |
| Complete revascularization | 0.80 (0.77, 0.83) | <0.001 |

S1 Table. Univariate predictors of survival.

PCI: percutaneous coronary intervention; LVEF: left ventricular ejection fraction; OnPCABG: on pumop coronary artery bypass grafts.

S2 Table. Test of proportional hazards assumption for gender

|  | rho | p |
| --- | --- | --- |
| Gender (all time-period) | -0.03706 | 0.0007 |
| Gender (1 year landmark) | -0.05080 and -0.00101 | 0.0544 and 0.9332 |

For Gender (1 year landmark) rho and p values are for One-year and over one-year landmark analysis respectively.


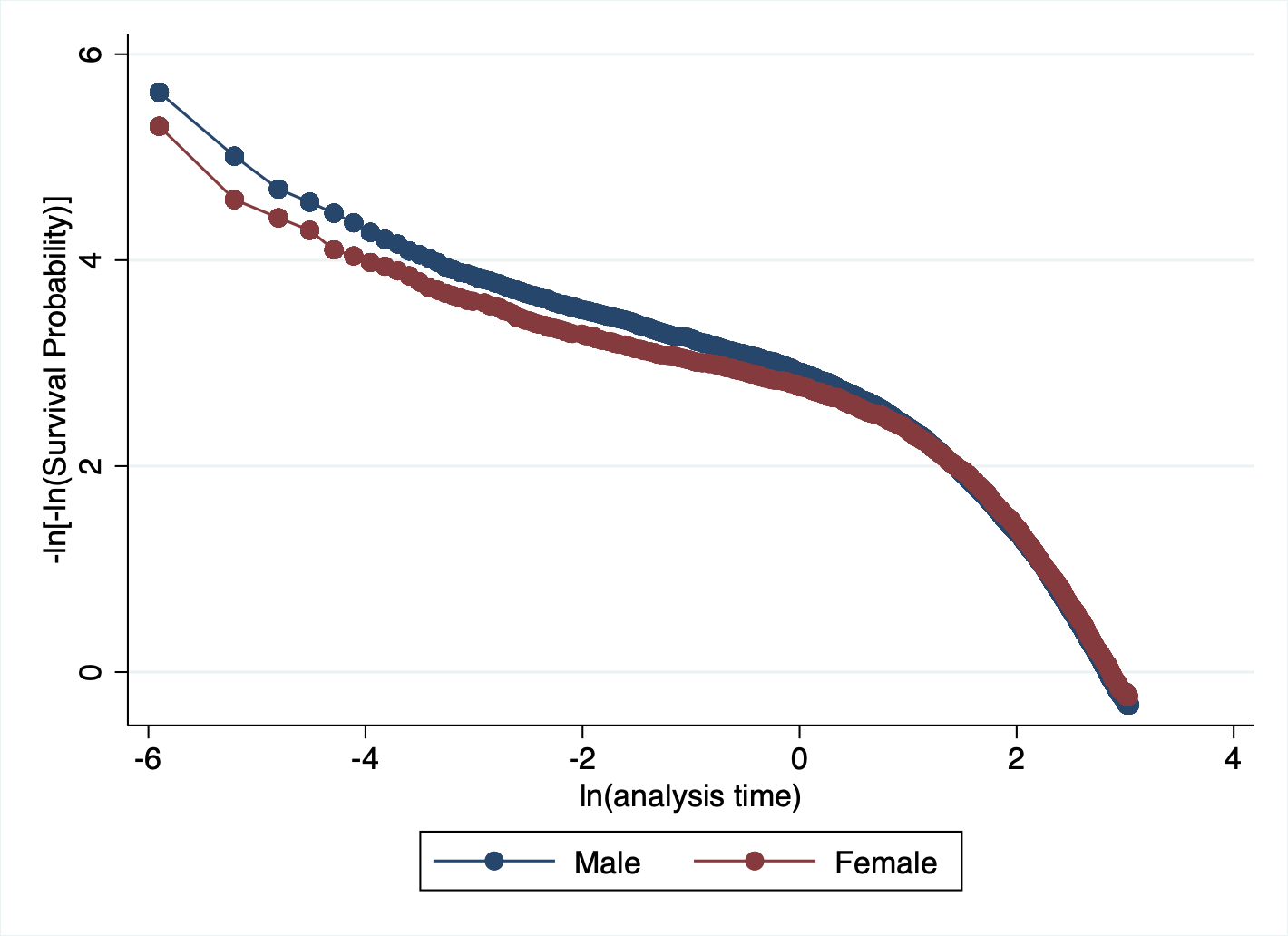


S1 Figure. Log-log proportional hazard assessment. Lines are not paralell which support violation of PH assumption.

Figure S2. Overall survival A) Before 2013 (p=0.0259) and B) After 2013 (p=0.0288).

S3 Figure. Forest plot of subgroup analysis on the impact of gender on survival.
